# Automated Protocoling for MRI Exams—Challenges and Solutions

**DOI:** 10.1007/s10278-022-00610-1

**Published:** 2022-08-30

**Authors:** Jonas Denck, Oliver Haas, Jens Guehring, Andreas Maier, Eva Rothgang

**Affiliations:** 1 Pattern Recognition Lab, Department of Computer Science, Friedrich-Alexander Universität Erlangen-Nürnberg, Erlangen, Germany; 2 Department of Industrial Engineering and Health, Technical University of Applied Sciences Amberg-Weiden, Weiden, Germany; 3 Siemens Healthcare GmbH, Erlangen, Germany

**Keywords:** Machine learning, Magnetic resonance imaging, Automated protocoling, Automated order entry

## Abstract

Automated protocoling for MRI examinations is an amendable target for workflow automation with artificial intelligence. However, there are still challenges to overcome for a successful and robust approach. These challenges are outlined and analyzed in this work. Through a literature review, we analyzed limitations of currently published approaches for automated protocoling. Then, we assessed these limitations quantitatively based on data from a private radiology practice. For this, we assessed the information content provided by the clinical indication by computing the overlap coefficients for the sets of ICD-10-coded admitting diagnoses of different MRI protocols. Additionally, we assessed the heterogeneity of protocol trees from three different MRI scanners based on the overlap coefficient, on MRI protocol and sequence level. Additionally, we applied sequence name standardization to demonstrate its effect on the heterogeneity assessment, i.e., the overlap coefficient, of different protocol trees. The overlap coefficient for the set of ICD-10-coded admitting diagnoses for different protocols ranges from 0.14 to 0.56 for brain/head MRI exams and 0.04 to 0.57 for spine exams. The overlap coefficient across the set of sequences used at two different scanners increases when applying sequence name standardization (from 0.81/0.86 to 0.93). Automated protocoling for MRI examinations has the potential to reduce the workload for radiologists. However, an automated protocoling approach cannot be solely based on admitting diagnosis as it does not provide sufficient information. Moreover, sequence name standardization increases the overlap coefficient across the set of sequences used at different scanners and therefore facilitates transfer learning.

## Introduction

Although the growth of medical imaging utilization has slowed down in comparison with other medical services, radiology services remain crucial for the diagnosis and treatment of many diseases [1, 2]. Claims of overutilization of medical imaging and a high contribution of radiology services to overall healthcare expenditures have led to medical imaging’s growth slowdown [3]. However, the contribution of medical imaging to overall healthcare expenditures remains significant. Consequently, recent discussions have focused on increasing the efficiency and value of medical imaging in general [2] and of specific imaging modalities, such as magnetic resonance imaging (MRI) in particular [4]. Approaches to increase the value of MRI include the development of abbreviated MRI protocols [5, 6], as well as new imaging techniques to accelerate imaging acquisition and increase the diagnostic value. Moreover, artificial intelligence (AI) plays a vital role in developing enhanced imaging techniques and diagnosis support for the radiologist. AI has shown potential to accelerate imaging [7], supports the radiologists during imaging interpretation [8], and is subject to ongoing research to increase efficiency further [9].

Furthermore, the potential of AI applications beyond imaging acquisition and interpretation has already been recognized [10]. It includes, but is not limited to, creating and selecting study protocols, optimizing MR scanner utilization, scheduling, and automated billing [11]. These applications constitute promising approaches to optimize the clinical workflow, reduce the workload for clinical staff and radiologists, and lower costs. It enables radiologists to focus on image interpretation, patient care, and communication.

However, some of these methods have not reached acceptable performance yet to automate sub-tasks of the clinical workflow fully, which can limit the benefit for the clinical practice.

This is also true for study protocol selection for MRI exams (protocoling). Protocoling describes the process of selecting an adequate imaging protocol under consideration of the ordered procedure, clinical indication, and medical history. A set of pre-defined, institution-specific protocols for each imaging modality is in general available for selection that is listed in the protocol tree of the scanner. Each protocol typically consists of multiple MR pulse sequences, which are parameterized through sequence parameters that determine image contrast, resolution, signal-to-noise ratio, and/or scan time. The parameterized pulse sequences are identified through a user-defined sequence name that describes the resulting contrast and additional information (imaging orientation, pre-/post-contrast, slice thickness, etc.).

Pulse sequences are often used for imaging of multiple body parts and use cases (e.g., turbo/fast spin echo sequences [12] ) or can be used almost exclusively for a single body region (e.g., cine sequences used for cardiac MRI [13] or diffusion tensor imaging used for brain MRI [14] ). This increases the amount and variability of MR sequences at a scanner and protocoling complexity.

Although protocoling is recognized as an amendable target for workflow automation through the application of AI [15], and efforts are undertaken to automate protocoling (see Section “Literature Review”), published results have several limitations that diminish workflow benefits. These limitations are outlined, analyzed, and quantified in this work.

First, we describe the clinical MRI workflow, including critical workflow steps and potential pitfalls for protocol selection. Then, we review current literature on automated protocoling for MRI exams and perform a data-driven analysis to describe challenges for an AI-based automated protocoling approach for MRI and propose solutions for these challenges.

Our contributions are as follows:

- We provide in-depth analysis of the latest research on automated protocoling for MRI exams and describe common obstacles (information availability, MRI protocol comparability) that restrict its integration into the clinical workflow.
- We analyze the patient’s admitting diagnosis mapped to the assigned MRI protocol and assess the consistency of the mapping.
- We assess the heterogeneity of protocol trees on protocol and sequence level and outline associated problems complicating a robust automated protocoling approach.
- We describe potential solutions to these challenges for successful automated protocoling for MRI exams.

## Background

### MRI Workflow Description

First, we want to provide a general overview of the clinical workflow for an MRI exam and describe the critical decision steps that impact correct protocoling. The first step for any MRI exam is the referring physician’s procedure order through the transmission of an ordered procedure and the reason for the MRI exam (i.e., clinical indication). The procedure order may require the consultation of a clinical decision support (CDS) system to determine the necessity and appropriateness of the ordering [16].

An insufficient clinical indication or the wrong ordered procedure can be the first source of errors for protocoling. If different MRI systems are available (e.g., 1.5 T and 3 T systems), the schedulers allocate a time slot at the appropriate system for the patient exam. This may be subject to the radiology practice/department’s site conditions as some procedures may only be performed at a specific system, e.g., dedicated systems for a certain body region. The responsible radiologist selects the appropriate protocol depending on the clinical indication, the ordered procedure, prior imaging exams and radiology reports, medical history in the electronic medical record (EMR), and relevant lab results [17]. The technologist performing the exam must select the correct protocol on the MRI host computer as specified by the radiologist. Following exam completion, the radiologist interprets the images and makes a diagnosis. Then, the administrative staff completes documentation and the results are communicated to the referring clinician and the patient.

**Fig. 1:**
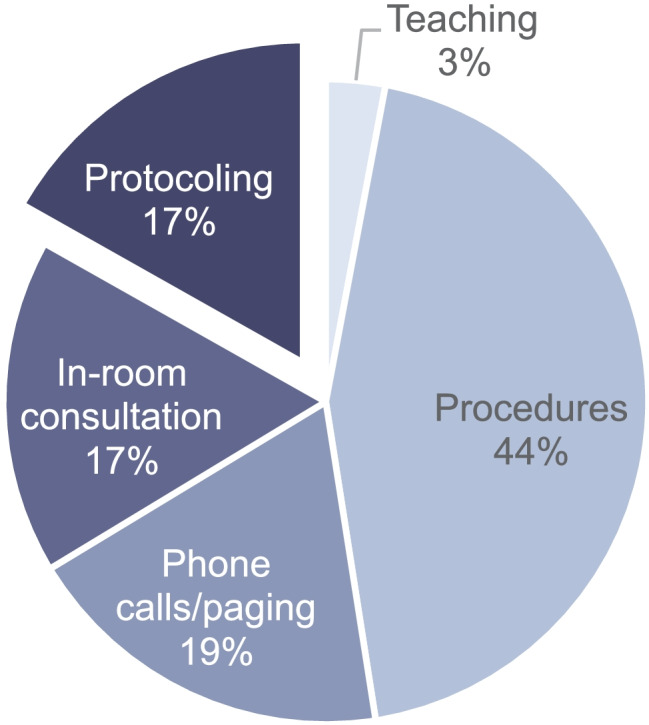
Timeshare spent by radiologists on different non-image-interpretive tasks [18]

Protocoling is a time-consuming task and prone to errors [18, 19]. However, it is a crucial workflow step as incorrect protocoling can diminish the clinical value of the subsequent MRI exam. In a workflow observation study, Schemmel et al. measured radiologists’ activities in an academic neuroradiology practice [18]. Within non-image-interpretative tasks (procedures, phone calls, in-room consultation, protocoling, teaching) of radiologists, 17% of their time is spent on protocoling, therefore constituting a significant share of their daily workload (see Fig. 1).

Moreover, Ginat et al. analyzed MRI protocoling errors in the neuroradiology department of a hospital-based academic medical center for 4,244 MRI exams within a 6-month period [19]. In total, 140 protocoling-related issues occurred. They categorized these issues into scheduling errors (1.5 T vs. 3 T), ordering clinician errors (e.g., wrong body region), trainee errors (omitting a crucial sequence), technologist errors (missed pre-processing), and inherent protocol problems (e.g., insufficient field-of-view). 37.1% of the reported errors (scheduling, ordering clinician, trainee errors) are amendable through protocoling automation and workflow improvements.

In a former study [20], the impact of missing information within the clinical indication was assessed by evaluating the concordance and completeness of clinical indications in order requisitions compared to provider notes, which offer a more comprehensive picture of the patient’s condition. Indications were incomplete in 81% and discordant in 42% within 315 assessed MRI and CT exams. This has a potential impact on correct protocol selection in 8% and on exam interpretation in 43% of the cases.

Protocoling forms the basis for the patient’s diagnosis and further examinations [20]. With an improper protocol, the radiologist might not interpret the MR images accurately, which could lead to patient harm in the worst case. Less severe consequences of incorrect protocoling include patient recalls [21] and therefore additional, avoidable costs for the healthcare system. Consequently, every protocoling error must be avoided.

Besides the benefit of avoiding protocoling errors with their associated consequences, automating protocoling can result in a significant reduction of the workload for radiologists that can be spent on image interpretation, patient care, and communication. Consequently, automated protocoling is anticipated to enhance the clinical MRI workflow considerably.

### Literature Review

Different machine learning approaches for automated protocoling of MRI exams have been developed recently, with differences in the underlying machine learning task, input data, and achieved performance, presented in the following (see Table 1).

**Table 1:** Publication overview on automated protocoling for MRI exams

Ref	Task	Classification type	Method	Input data	Dataset size (training/test)	Accuracy^1^
[22]	(1) Protocol selection from 13 MRI brain protocols (2) Contrast administration assignment (3) Priority determination	(1) Multi-class (2) Binary (3) Binary	TDM + random forest	Clinical indication	11,185/2,797	(1) 82.9% (2) 83.0% (3) 88.2%
[23]	Sequence selection from 41 MRI sequences	Multi-label	TDM + gradient boosting machine	Clinical indication, patient age, gender, study type	5,240/2,246	95.1%
[24]	Protocol selection from 14 abdomen/pelvis MRI protocols	Multi-class	IBM Watson	Clinical indication	253/82	93%
[25]	Contrast administration assignment for MSK MRI protocols	Binary	IBM Watson	Clinical indication	1,240/280	83.2%
[26]	Protocol selection from 17 MSK routine and tumor MRI protocols	Multi-class	CNN	Referring department, body region, contrast administration, patient age, gender	5,258/1,018	94.2%
[27]	Protocol selection from 400 CT and MRI protocols	Multi-class	Word2Vec + neural network	Clinical indication Free-text comments Requested procedure	263,700/29,300	72%
[28]	Protocol selection from 108 CT and MRI protocols	Multi-class	Bag of words + neural network	Clinical indication and history	14,519/3,673	84%
[29]	Protocol selection from 88 CT and 293 MRI protocols	Multi-class	TF-IDF + SVM	Requested procedure, department, anatomy, patient history	166,534/41,634 (CT) 392,771/ 98,193 (MRI)	92.2% (CT) 86.9% (MRI)
[30]	Protocol selection from 8 MRI spine and 9 brain protocols	Multi-class	fastText^2^ + XGBoost^3^	Clinical indication	990/151 (spine) 6,067/1,071 (brain)	83.4% (spine) 85.4% (brain)
[31]	Protocol selection from 200/93/48 MRI protocols	Multi-class	One-hot-encoding + neural network	Clinical indication	81,356/34,868	82.8%, 73.8%, 69.3%^4^

Legend: Method: ^a^*TDM*, term-document matrix; ^b^*CNN*, convolutional neural network; ^c^*TF-IDF*, term frequency–inverse document frequency; ^d^*SVM*, support vector machine. Classification type: ^e^Binary: assignment of a binary label (0/1) to a single target variable, e.g., administer contrast medium or not; ^f^Multi-class: classification of an instance into one of more than two categories, e.g., select one protocol out of *n* (*n* > 2); ^g^Multi-label: classification of an instance of *n* different binary labels, e.g., select *n* MRI sequences for a certain MRI protocol

^1^Referring to the top 1 accuracy. Multiple works also evaluated the top 5 accuracy scores

^2^fastText: library for learning of word embeddings (https://fasttext.cc/, accessed 7th November 2021)

^3^XGBoost: library for gradient boosting (https://github.com/dmlc/xgboost, accessed 7th November 2021)

^4^The accuracy scores refer to multiple evaluated protocol subsets

Brown and Marotta [22] developed three natural language processing (NLP) models that were trained on free-text clinical indications transformed into a term-document matrix, patient and referral demographics to predict the selection of the correct brain MRI protocol (task 1), to evaluate the need for contrast administration (task 2), and to determine priority (task 3) and achieved an accuracy of 82.9%, 83.0%, and 88.2% for tasks 1, 2, and 3.

In another study [23], the authors extended their former study by training a machine learning model to select MRI sequences for the MRI exam based on the input given by patient age and gender, study type, and the study’s clinical indication. They used data from a single academic hospital site, where radiologists could choose from 41 different MRI sequences. They reached an accuracy of 95.1% using a gradient boosting machine and state that performing predictions at sequence level allows a more robust clinical application.

IBM Watson was used to predict abdomen/pelvis MRI protocols based on free-text clinical indications [24] and to predict the need for intravenous contrast for musculoskeletal MRI protocols based on free-text clinical indications [25]. They reported an overall accuracy of 83.2% on their test set of 280 cases. However, with respect to a second reader’s contrast assignment, who only had access to the clinical indication (without any further information, such as the requested study type), the classifier based on IBM Watson achieved 88.6%. This performance discrepancy shows that the clinical indication alone is sometimes insufficient for determining the need for contrast agent for MRI exams.

A neural network was trained with the word embedding of the clinical indication obtained with Word2vec combined with the requested exam type to predict MRI and CT studies [32, 33]. The network correctly predicted the most likely protocol from over 400 different protocols in 72% and the “top 3” protocol in 90% of the test cases. The error analysis revealed that a large subset of prediction errors was due to data points with minimal clinical, i.e., insufficient information.

The published approaches all use the clinical indication as input as it is the most discriminative feature for protocol selection. In addition to the clinical indication, multiple studies extend the input data by patient demographics, requested procedure, or additional free-text comments. Moreover, different machine learning algorithms have been applied, whereas no specific machine learning model or approach has prevailed and showed superior performance.

Although the presented studies vary in their algorithmic approach, used input data, and achieved performance, they share limitations restricting their use in the daily clinical workflow. In the following, we outline and analyze these limitations.

## Materials and Methods

In the first part of our work, we assess the information content the clinical indication offers for the protocol selection. Therefore, we analyzed the mapping of ICD-10-coded admitting diagnosis to MRI protocol and computed the overlap of admitting diagnoses across MRI protocols.

Another challenge for automated protocoling is the heterogeneity of different protocol trees that complicate applying one trained model to a new scanner/protocol tree. We demonstrate that by assessing the protocol and sequence similarity between different protocol trees in the second part of our work.

Little research has been published on the heterogeneity/similarity of MRI protocol trees with respect to the set of used protocols and sequences [11, 34]. In general, it is difficult to qualitatively compare protocol trees from different scanner types and sites due to the various considerations that must be taken into account for protocol tree configuration. Furthermore, an optimal MRI protocol tree setup can hardly be defined, since external and internal guidelines, types of exams, and application availability (e.g., sequences) vary significantly and change frequently.

However, the similarity of protocol trees can be assessed quantitively to measure protocol and sequence overlap as a proxy for standardization and harmonization. The degree of similarity of different protocol trees directly impacts whether an automated protocoling approach is transferable, without retraining or fine-tuning, to a new scanner or site.

The methods and used data are described in the following in more detail.

### Set Similarity Metrics

For the similarity assessment between the set of admitting diagnoses for different protocols (“Admitting Diagnosis Protocol Overlap”) and the set of protocols and sequences used across scanners (“Protocol Tree Assessment”), a suitable similarity metric must be employed. Widely used similarity metrics for two finite sample sets $A$ and $B$ are the Jaccard similarity coefficient (also known as Jaccard index) and the overlap coefficient. The Jaccard index is defined as follows:

$$J(A,B)=\frac{\left|A\cap B\right|}{\left|A\cup B\right|}$$

The overlap coefficient ($oc$) is related to the Jaccard similarity coefficient and defined as follows:

$$oc(A, B) =\frac{\left|A\cap B\right|}{min(\left|A\right|,\left|B\right|)}$$

However, while both coefficients measure the overlap between two sets, only the overlap coefficient is sensitive to the sizes of the sets. If $A$ is a subset of $B$ (or vice versa), the overlap coefficient is 1.

The overlap coefficient has to be proven to be better suited for small datasets and in particular for different sized sets than the Jaccard index [35].

### Admitting Diagnosis Protocol Overlap

We evaluated how consistently the same MRI protocols are assigned to patients with the same clinical indication by computing the overlap coefficient for different protocols with respect to the set of admitting diagnosis ICD-10 codes. This allows us to assess the information share provided by the admitting diagnosis (i.e., clinical indication) for the protocol decision. We used the admitting diagnosis ICD-10 codes since, unlike the free-text clinical indication, the ICD-10-coded admitting diagnosis constitutes a data basis that enables categorization and comparison of clinical indications. The admitting diagnosis is usually coded during the documentation workflow step and can comprise one or more ICD-10 codes. It corresponds to the referring physician’s free-text clinical indication. Studies evaluating medical coding accuracy show that medical coding data serves as a reliable database for this research [36].

We computed the overlap coefficient with respect to the admitting diagnosis ICD-10 codes for all protocols within a certain body region and the complement overlap coefficient for each protocol. The complement overlap coefficient describes the set of admitting diagnosis ICD-10 codes for a single protocol (denoted by $A$) with respect to any other protocol in the dataset. More formally, the complement overlap coefficient is denoted by $oc\left(A, {A}^{c}\right)$, where ${A}^{c}$ is the complement of $A$: ${A}^{c}=\left\{x\in U| x\notin A\right\}$, with $U$ containing all elements within the (filtered) dataset.

We have compiled a dataset that comprises 2,106 MRI exams across all body regions from three MRI scanners of a single radiology practice (Zwanger-Pesiri Radiology, New York, USA). It contains the ICD-10-coded admitting diagnoses extracted from the institution’s radiology information system and MRI scanner log data [11] that contain information about the MRI exam (performed sequences, acquisition parameters, protocol name). The evaluated data comprise all MRI exams performed with the three scanners within January and June 2019. The data were stripped of any patient-identifiable information.

### Protocol Tree Assessment

We also analyzed challenges associated with the heterogeneity of protocols (protocol trees) and assessed the similarity between different protocol trees on protocol and sequence level. Therefore, we computed the overlap coefficient for different protocol trees (for all body regions) and the sequences used within the protocols. However, since the sequence name can be adapted individually, it is not suitable for the objective characterization of a protocol step, i.e., the parameterized MRI sequence. Therefore, different strategies were recently developed to categorize and standardize MRI sequence descriptions, which can be used as a meaningful, standardized feature for machine learning applications [11, 37, 38]. We used a heuristic rule–based approach [11] that generates a standardized sequence name based on the MR acquisition parameters of a sequence (e.g., TR, TE, imaging technique, imaging orientation). We show the effect and benefit of sequence name standardization on assessing sequence heterogeneity across different protocol trees.

We evaluated the protocols from three different MRI scanners of the same radiology practice. Two scanners (scanner 1 and 2) are located at the same site, and one scanner (scanner 3) is located at a different site. All MRI scanners are 3 T MAGNETOM Skyra systems (Siemens Healthcare, Erlangen, Germany) with the same software baseline.

## Results

### Admitting Diagnosis Protocol Overlap

We analyzed the overlap of admitting diagnosis ICD-10 codes within MRI exams of two selected body regions, brain/head and spine, as MRI is frequently used for various use cases for these body regions. We only incorporated protocols that were performed more than five times within our dataset.

The average number of admitting diagnosis ICD-10 codes per exam is low for both spine (1.1) and brain/head (1.2), and single code occurrence ranges from 1 to 182 for spine and to 42 for brain/head exams, respectively (Table 2).

**Table 2:** Data properties of the evaluated MRI exams for the selected body regions

Body region	Spine	Brain/head
Number of exams	727	249
Number of unique ICD-10 codes	127	98
Label cardinality	1.1	1.2
Label density	0.01	0.01
Label diversity (code sets)	177	118

Legend: Label cardinality: average number of admitting diagnosis ICD-10 codes per exam; Label density: cardinality divided by the number of unique codes; Label diversity: number of distinct code sets

Since the frequency of the protocols varies widely within the dataset (ranging from 6 to 341), we computed the overlap coefficient to assess the similarity of admitting diagnosis and protocol mapping.

**Fig. 2:**
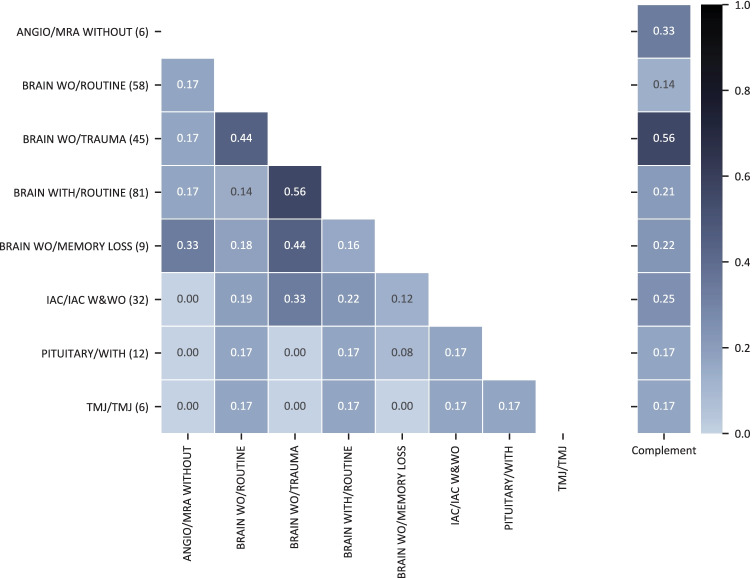
Heat map visualization of the overlap coefficient of admitting diagnosis ICD-10 codes between MRI head protocols. The annotated decimal values describe the overlap coefficient for the protocol specified on the ***y***-axis and the protocol specified on the ***x***-axis. The last column denotes the complement overlap coefficient for the protocol specified on the ***y***-axis. Abbreviations:—WITHOUT: no contrast medium is administered. WITH means images were acquired after the administration of contrast medium only. W&WO stands for “with and without” contrast medium. IAC stands for internal auditory canal and TMJ for temporomandibular joint

**Fig. 3:**
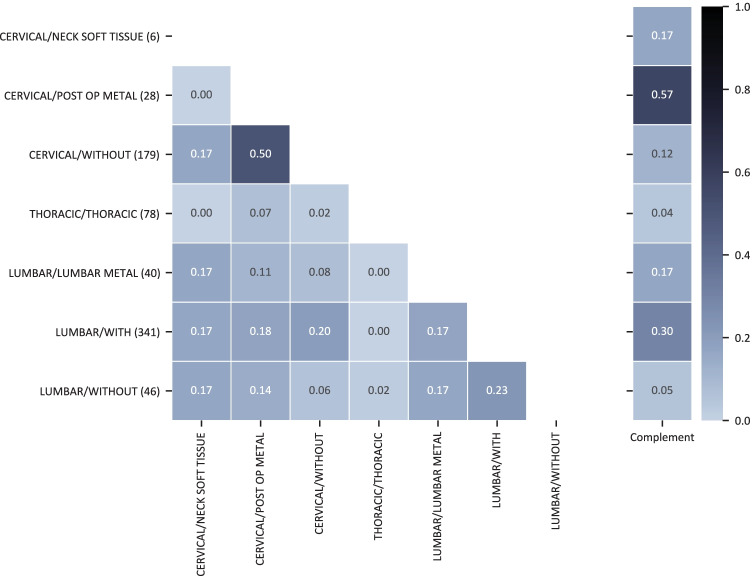
Heat map visualization of the overlap coefficient of admitting diagnosis ICD-10 codes between MRI spine protocols. The annotated decimal values describe the overlap coefficient for the protocol specified on the *y*-axis and the protocol specified on the *x*-axis. The last column denotes the complement overlap coefficient for the protocol specified on the *y*-axis

Figures 2 and 3 visualize the overlap coefficients for the set of admitting diagnosis codes associated with the different brain/head and spine MRI protocols, respectively. As the overlap coefficient is symmetric, i.e., $oc\left(A,B\right)= oc(B, A)$, so is the heatmap. Note that $A$ and $B$ comprise here the set of admitting diagnosis code combinations for two different MRI protocols. Thus, an element within these sets can consist of single or multiple ICD-10 codes.

The overlap coefficient with respect to the complement ranges from 0.14 to 0.56 for brain/head MRI protocols and 0.04 to 0.57 for spine protocols. When comparing single protocols, the $oc$ ranges from 0 to 0.56 and 0 to 0.5 for brain/head and spine exams, respectively.

The decision between two protocols can solely be based on the admitting diagnosis only if the overlap coefficient is 0; i.e., no overlap of the associated admitting diagnoses with other protocols exists. Consequently, the lower the overlap coefficient, the better for an automated protocol decision based on the admitting diagnosis.

### Protocol Tree Assessment

We have assessed protocol trees from three scanners of the same model (MAGNETOM Skyra) with the same software baseline, from the same radiology practice, but from two different sites. This allows a valid quantitative comparison of protocol and sequence content and overlap.

On average, 103 protocols are used on each scanner, compiled from 209 different sequence names (across all body regions). MRI sequence categorization and standardization have the advantage that they reduce the variability of sequence descriptions significantly ($P<0.05$; chi-square test), and sequences become comparable across scanners and sites. When applying sequence name standardization to determine the sequence name solely based on the sequence parameter values, the number of unique sequence names is reduced to 97 on average. In Table 3, the differences in sequence content and protocol naming between the scanners are summarized.

**Table 3:** Quantitative assessment of the number of different protocols and sequences within the complete protocol trees used at three scanners of the same radiology practice. The overlap coefficient for (standardized) sequence and protocol names is computed with respect to scanner 1

		Scanner 1 (site A)	Scanner 2 (site A)	Scanner 3 (site B)
#	Unique sequence names	231	200	196
	Unique standardized sequence names	104	98	89
	Unique protocol names	107	103	100
$oc$	Sequence names	-	0.86	0.81
	Standardized sequence names	-	0.93	0.93
	Protocol names	-	0.83	0.79

A different set of sequence and protocol names are used on each scanner, with a decreased overlap coefficient for scanners at different sites compared to scanners at the same site. Applying sequence name standardization increases [13] the overlap for the set of sequence names is significantly ($P<0.05$). Thus, sequence name standardization demonstrates that the difference does not only arise from different sequence content but also from different or inconsistent sequence naming.

The common, i.e., overlapping, set of sequence and protocol names decreases significantly between scanners of the same and different sites. Consequently, an automated protocoling approach on data from one scanner/site will not be directly applicable to another scanner’s protocol tree, as a different set of sequences requires retraining or fine-tuning.

## Discussion

### Admitting Diagnosis Protocol Overlap

Through the analysis of the ICD-10-coded admitting diagnosis and the assigned MRI protocol, we showed that MRI protocol selection could not be done reliably on the admitting diagnosis only.

For example, the $oc$ for the spine protocols “CERVICAL/WITHOUT” and “CERVICAL/POST OP METAL” is 0.5; i.e., 50% of the admitting diagnoses associated with “CERVICAL/WITHOUT” were also associated with the “CERVICAL/POST OP METAL” protocol. In this case, crucial information missing is whether metal implants are present in the spine that is not included in the admitting diagnosis. On the other hand, the $oc$ for the spine protocols “LUMBAR/WITHOUT” and “LUMBAR/LUMBAR METAL” is significantly lower (0.17). Inconsistencies in protocoling, additional clinical information, or the fact that the protocol name does not reflect the complete application range can lead to these differences.

The presented examples show that multiple protocols for the same admitting diagnosis may be a suitable protocol choice, and more information than only the admitting diagnosis (and consequently also the clinical indication) needs to be assessed to be able to make an informed protocol decision [25]. Selecting the protocol from the clinical indication only could lead to patient harm as contraindications for MRI (e.g., allergies, metal implants, pregnancies) [39] may be overseen, or with less severe consequences, a suboptimal protocol may be selected, and image interpretation may be impeded.

Similar results were obtained for the determination of contrast medium administration [25], as a radiologist assessing the need for contrast medium solely based on the clinical indication achieved only 89.6% accuracy (251/280 cases). Another study identified a significant impact of incomplete exam indications on correct protocoling by analyzing provider notes and clinical indications [20].

However, limitations within our study exist. Although the ICD-10-coded admitting diagnosis does not necessarily reflect the complete clinical indication as information can be lost through coding, it is a useful indicator to assess the information content provided by the clinical indication for the protocol decision. Moreover, it may serve as a systematic approach for protocol configuration by minimizing the overlap with respect to the admitting diagnosis or simply identifying redundant, i.e., overlapping, protocols.

Although our analysis is based on data (2,106 cases) from multiple scanners and sites, it is limited to data from a single radiology practice. Validation of our results with data from additional radiology departments or practices is subject to future work.

### Protocol Tree Assessment

In the second part of our work, we computed the overlap coefficient for the set of protocols and sequences used at different MRI scanners. Moreover, we showed that standardized sequence representations lead to a significant reduction in feature dimensionality across scanners of the same and different sites.

As MRI protocols are usually adapted regularly to reduce protocol creep [34], to be aligned with updated guidelines or after a scanner software update, a comprehensive automated protocoling approach has to cope with protocol changes. However, an automated protocoling approach that has learned to assign the correct protocol name [22, 25, 26] must be retrained whenever new protocols are added or existing protocols are adapted. Therefore, developing a machine learning system to predict required MRI sequences rather than the textual representation of an MRI protocol (i.e., the site-specific protocol name) is crucial to cope with protocol variation and changes and is anticipated to lead to a more robust application. Furthermore, the robustness of an automated protocoling model can be improved with sequence name standardization, which leads to a reduced feature space and makes the approach independent of the user-given sequence name. Despite the protocol heterogeneity of different protocol trees, the aggregation of a large dataset may help to learn different protocoling setups for a certain clinical question and may increase the performance of machine learning models for automated protocoling in future works.

## Conclusion

Automated protocoling for MRI exams has the potential to reduce the workload for radiologists and prevent protocoling errors that may diminish the diagnostic value of an MRI exam and lead to re-scans. Unfortunately, recently published approaches have not reached an acceptable performance yet to automate protocol assignment for MRI exams fully and rely only on the clinical indication for the prediction. However, an automated protocoling approach cannot be solely based on the admitting diagnosis as it often does not provide sufficient information for the distinction between two suitable protocols. Moreover, the heterogeneity between protocol trees hinders the deployment of a trained machine learning model to the protocol tree of a new scanner. Sequence name standardization increases the overlap coefficient across the set of sequences used at different scanners and can be crucial to increase the robustness of automated protocoling approaches.

## Data Availability

Private dataset.

## Code Availability

Custom code.
